# Maize Benefits the Predatory Beetle, *Propylea japonica* (Thunberg), to Provide Potential to Enhance Biological Control for Aphids in Cotton

**DOI:** 10.1371/journal.pone.0044379

**Published:** 2012-09-12

**Authors:** Fang Ouyang, Xingyuan Men, Bing Yang, Jianwei Su, Yongsheng Zhang, Zihua Zhao, Feng Ge

**Affiliations:** 1 State Key Laboratory of Integrated Management of Pest Insects and Rodents, Institute of Zoology, Chinese Academy of Sciences, Beijing, People’s Republic of China; 2 Institute of Plant Protection, Shandong Academy of Agricultural Sciences, Jinan, People’s Republic of China; 3 College of Bio-Safety Science and Technology, Hunan Agricultural University, Changsha, People’s Republic of China; TGen, United States of America

## Abstract

**Background:**

Biological control provided by natural enemies play an important role in integrated pest management. Generalist insect predators provide an important biological service in the regulation of agricultural insect pests. Our goal is to understand the explicit process of oviposition preference, habitat selection and feeding behavior of predators in farmland ecosystem consisting of multiple crops, which is central to devising and delivering an integrated pest management program.

**Methodology:**

The hypotheses was that maize can serve as habitat for natural enemies and benefits predators to provide potential to enhance biological control for pest insects in cotton. This explicit process of a predatory beetle, *Propylea japonica*, in agricultural ecosystem composed of cotton and maize were examined by field investigation and stable carbon isotope analysis during 2008–2010.

**Principal Finding:**

Field investigation showed that *P. japonica* adults will search host plants for high prey abundance before laying eggs, indicating indirectly that *P. japonica* adults prefer to inhabit maize plants and travel to cotton plants to actively prey on aphids. The δ^13^C values of adult *P. japonica* in a dietary shift experiment found that individual beetles were shifting from a C_3_- to a C_4_-based diet of aphids reared on maize or cotton, respectively, and began to reflect the isotope ratio of their new C_4_ resources within one week. Approximately 80–100% of the diet of *P. japonica* adults in maize originated from a C_3_-based resource in June, July and August, while approximately 80% of the diet originated from a C_4_-based resource in September.

**Conclusion/Significance:**

Results suggest that maize can serve as a habitat or refuge source for the predatory beetle, *P. japonica*, and benefits predators to provide potential to enhance biological control for insect pests in cotton.

## Introduction

Agricultural intensification has resulted in the simplification of agricultural landscapes through the expansion of agricultural land, enlargement of field size and removal of non-crop habitat, which has resulted in the rapid decline of farmland biodiversity and a concentration of the remaining biodiversity in the field edges and non-crop habitats [1], [2], [3]. The decline of biodiversity and the simplification of the landscape composition can affect the functioning of natural pest control because non-crop habitats provide refuge for a broad spectrum of natural enemies, and the exchange of natural enemies between crop and non-crop habitats is likely to be diminished in landscapes dominated by arable cropland [1]. An ecologically based strategy to address pest problems is the promotion of earlier or greater colonization by natural enemies through specific habitat management techniques [4], [5]. Many such strategies emphasize roles of non-crop habitats to enhance natural enemy populations, but little research has been directed toward the explicit process of oviposition preference, habitat selection and feeding behavior of natural enemies in agricultural systems consisting of multiple crops such as C_3_ and C_4_ plants.

Cotton and maize are important crops and provide the main agricultural landscape in Northern China (Ge Feng 1995). The cotton aphid, *Aphis gossypii* (Glover), is a serious sucking pest of cotton that can cause substantial yield loss [6]. Especially since the 1990s, transgenic Bt (*Bacillus thuringiensis*) cotton has become an important tool for insect pest management of cotton worldwide [7], [8], [9]. The decreased use of broad-spectrum pesticides for control of cotton bollworm in Bt cotton fields has resulted in increases in non-target populations of sucking insects, such as mirids, in multiple crops in China [10], [11]. Thus, Bt cotton is just one component to be considered in the overall management of insect pests in the diversified cropping systems common throughout China [9], [11]. Maize (*Zea mays* L.), a C_4_ plant, has been widely planted in China and occupied 29.9 million ha in 2008 [12]. Maize aphid, *Rhopalosiphum maidis* (Fitch), is a worldwide pest of maize. *Propylea japonica* is a prevalent mobile predator of aphids in maize and cotton and moves among crops in agricultural systems [13], [14], [15]. Much research on its predation on aphids in cotton has been reported [13], [14], [15]. However, the factors affecting intercrop movement and foraging behavior of *Propylea japonica* between cotton and maize remain to be elucidated from a landscape perspective.

Carbon isotope analysis is used to track habitat selection and feeding behavior of predators and parasitoids [5], and the dispersal abilities of phytophagous insects in agricultural landscapes composed of C_3_ and C_4_ plants [16]. The stable carbon isotope ratios (^13^C:^12^C) of was expressed as δ^13^C, a parts per thousand (‰) difference relative to a reference material. Carbon isotope values of plant species remain intact when transferred in the food chain to phytophagous insects and the predators or parasitoids that consume them [5], [16], [17], [18], [19]. This process can effectively mark plant species such as C_3_ and C_4_ plants, phytophagous insects and their natural enemies, whereas any switch from a C_3_- to a C_4_-based diet (or vice versa) causes a change in carbon isotope ratio close to the δ^13^C value of the recent diet [5], [17], [18], [20], [21]. Documentation of this has not been conducted for the lady beetle, *P. japonica* in agricultural systems consisting of cotton and maize plants.

In the current study, field investigation and stable carbon isotope ratio analysis (^13^C/^12^C) from complementary laboratory and field samples between 2008–2010 were used to examine the process of oviposition preference, crop colonization and subsequent feeding by the predatory beetle, *P. japonica* in agricultural landscapes composed of cotton and maize. In this agricultural landscape system, “landscape” was defined in a general sense, as a spatially heterogeneous area [22] that is scaled relevant to the process or organism under investigation [23]. The effect of landscape structure on predator–prey interactions in red clover was studied in experimental model landscape system [24]. Our objectives were to:

1. establish the oviposition preference of *P. japonica* at the patch and landscape levels within agricultural landscapes composed of cotton and maize;
2. identify the preferred crop patches of *P. japonica* adult populations to inhabit in agricultural landscapes composed of cotton and maize, and define responses in population density of *P. japonica* adults to spatial variation;
3. and determine the feeding behavior of *P. japonica* in multiple crop landscapes composed of cotton and maize.

## Materials and Methods

### Study Site and Experimental Design

**Figure 1:**
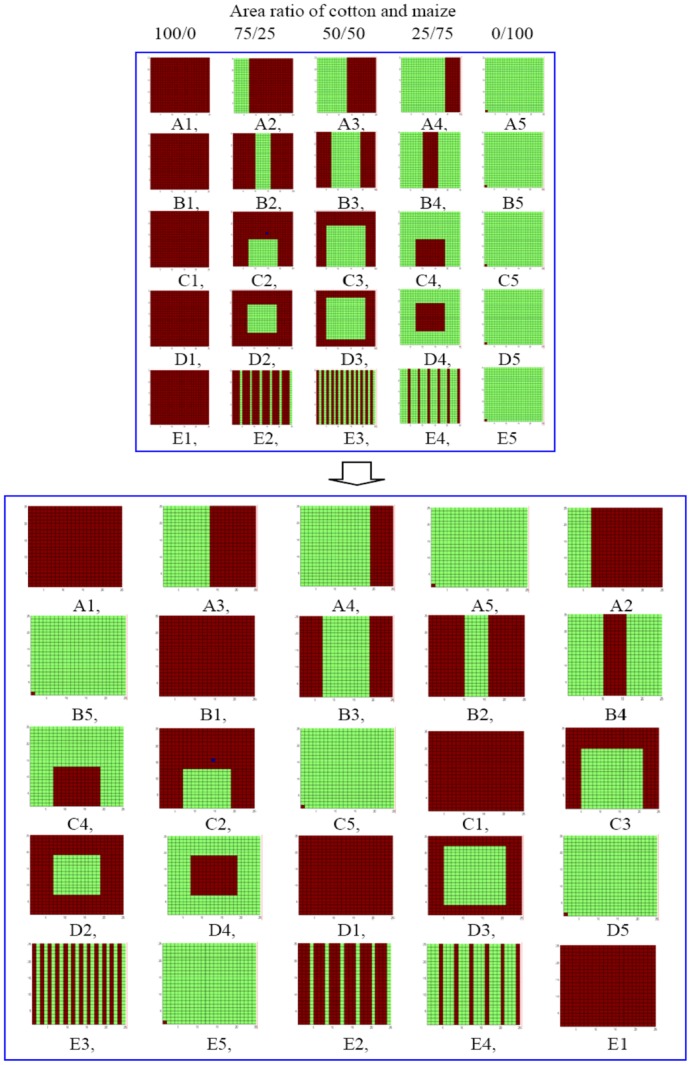
Spatial layout of the field experiment. The field was 90 m×90 m and divided into 25 15 m×15 m plots, each plot consisting of 24 rows and 50 columns. The spacing between neighboring plots was 3–4 m. Green and red areas in plot indicate the planting of cotton and maize.

The field experiment was performed during 2008–2010 and was conducted in a 1.2 ha field at the Langfang Experiment Station (39.53°N, 116.70°E) in the Hebei Province of China. Cotton and maize were the main crops in this station and accounted for a total area of ∼35 ha. An experimental model system was planed to study the explicit process of natural enemies in agricultural landscape system, and to define responses in population density of *P. japonica* adults to spatial variation. Plot treatments of spatial variation were based on patch area and spatial arrangements of cotton and maize. The experimental design in this study refer to the research described in the article of Crist, Pradhan-Devare *et al*. [25]. The experimental plot was 90×90 m and divided into 25 15 ×15 m plots, each plot consisting of 24 rows and 50 plants along a row. A 3 or 4-m gap was left between plots to buffer influence from arthropods in neighboring plots [26]. All vegetation between plots was removed, when necessary, to minimize effects from the surrounding environment. Plot treatments were based on the area ratio of cotton to maize and consisted of the following five proportions: 100/0 (A1, B1, C1, D1, E1),75/25 (A2, B2, C2, D2, E2), 50/50 (A3, B3, C3, D3, E3), 25/75 (A4, B4, C4, D4, E4), 0/100 (A5, B5, C5, D5, E5) (Fig. 1). Spatial arrangement were measured by landscape shape index(LSI), which equals the sum of the landscape boundary and all edge segments (*m*) within the landscape boundary involving the corresponding patch type (including borders), divided by the square root of the total landscape area (*m* ^2^), adjusted by a constant for a circular standard or square standard [27]. Linear regression was used to analysis the relationship between densities of *P. japonica* adults on maize and landscape shape index of plot. The field plots were arranged by randomly assigning treatments within rows and columns. Each plot in this field was defined as landscape plot, consisting of cotton patch and/or maize patch (Fig. 1).

Host plants used in this study were: (1) Bt cotton (GK12), a transgenic variety of cotton expressing the protein Cry1A and supplied by the Biotechnology Research Institute of the Chinese Academy of Agricultural Sciences (Beijing); and (2) maize (Jiyuan1), obtained from the Institute of Plant Protection, Chinese Academy of Agricultural Sciences (Beijing). The plant seeds were obtained from the Chinese Academy of Agricultural Sciences. Host plants used in the experiments were planted on April 28 in 2008, May 1 in 2009 and May 4 in 2010, and total planting consisted of ∼45,000 plants/ha grown without pesticides at the Langfang Experiment Station. Plants were watered as needed and fertilized with a controlled release fertilizer [28]. Total experimental area was ∼0.8 ha and equally divided between the two plants.

**Figure 2:**
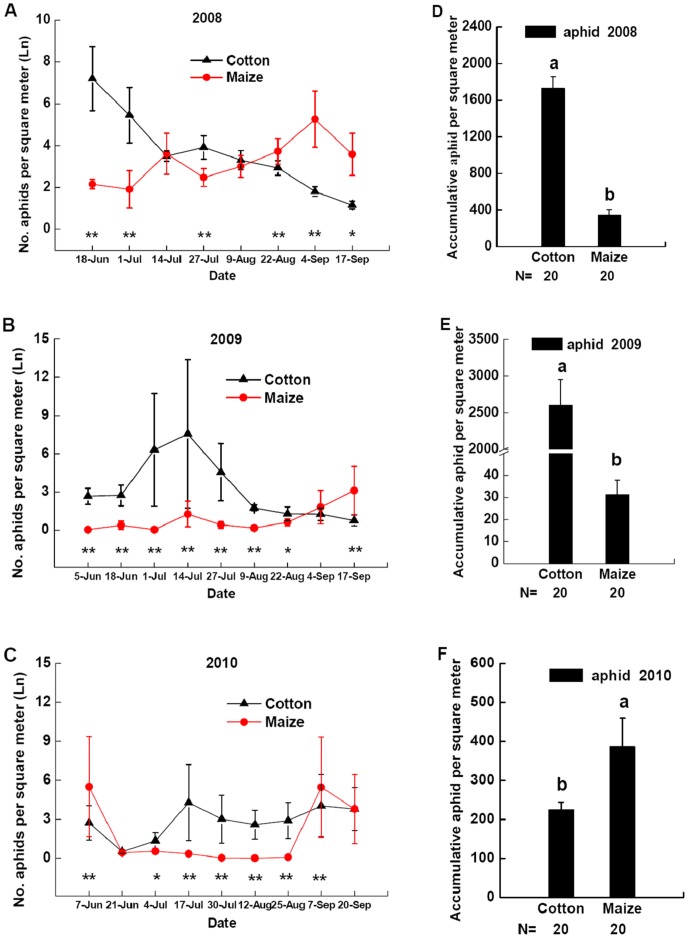
Dynamics of aphid density. Aphid density in cotton patches (black triangle) and maize patches (red circle) in field landscape plots in 2008 (**A**), 2009 (**B**), and 2010 (**C**). The data for aphid density were log-transformed (ln(n+1)). *Significant differences between densities of aphid in cotton patches and maize patches at p<0.05. **Significant differences between densities of aphid in cotton patches and maize patches at p<0.01. Densities of accumulative aphid in cotton patches and maize patches at all sample dates of field landscape plots in 2008 (**D**), 2009 (**E**) or 2010 (**F**). Different lowercases above the bars indicate significant differences in densities of accumulative aphid in cotton patches and maize patches at p<0.05. Data are presented per square meter of crop plants (mean±SE) with separate field landscape plots used as replicates. Sample size of cotton patch and maize patch are both 20. N indicates the size of samples tested.

### Insect Sampling and Collecting

The number of predatory beetles and aphids on cotton and maize were monitored in each experimental plot. All plots were sampled at ∼2-week intervals for a total of eight times in 2008, nine times in 2009 and nine times in 2010 from June 1 to September 30. Each plot was sampled by adopting a chessboard-like sampling method. And each time of sampling was from 7∶00 to 17∶00. Plants in every two rows and every three columns of a crop at each plot were sampled for eggs, larvae and adults of *P. japonica* and aphids by visual inspection with 80 person-minutes of effort expended per plot. The densities of predatory beetles and aphids on cotton and maize per square meter were determined by visually inspecting 60, 120, 180 or 240 plants per plot on the basis of treatment area and spatial arrangement.

**Figure 3:**
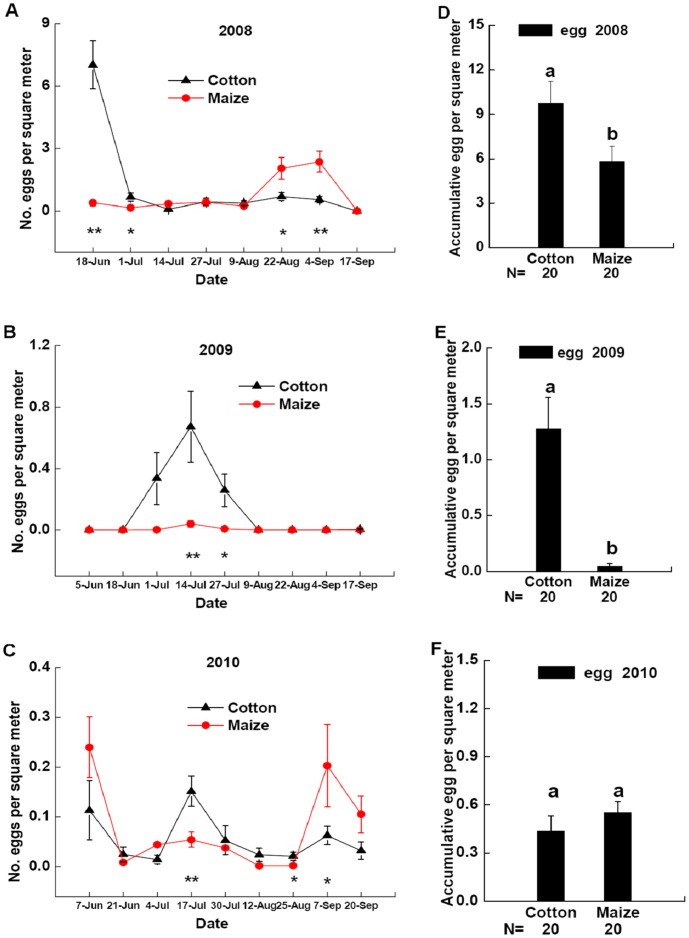
Dynamics of *P. japonica* eggs. Densities of *P. japonica* eggs in cotton patches (black triangle) and maize patches (red circle) in field landscape plots in 2008 (**A**), 2009 (**B**), and 2010 (**C**). *Significant differences between densities of *P. japonica* eggs in cotton patches and maize patches at p<0.05. **Significant differences between densities of *P. japonica* eggs in cotton patches and maize patches at p<0.01. Densities of accumulative *P. japonica* eggs in cotton patches and maize patches at all sample dates of field landscape plots in 2008 (**D**), 2009 (**E**) or 2010 (**F**). Different lowercases above the bars indicate significant differences in densities of accumulative *P. japonica* eggs in cotton patches and maize patches at p<0.05. Data are presented per square meter of crop plants (mean±SE) with separate field landscape plots used as replicates. Sample size of cotton patch and maize patch are both 20. N indicates the size of samples tested.

In addition to the monitoring efforts, *P. japonica* adults found on sampled plants in each plot were collected, labeled, placed in a plastic vial containing 95% ethanol and then stored in a freezer for preservation and analysis. Adult beetles were collected in the experimental field plots twice in 2008, four times in 2009 and eight times in 2010. Additionally, aphid and green leaf tissue samples from cotton and maize plants were also collected at each plot after monitoring.

**Figure 4:**
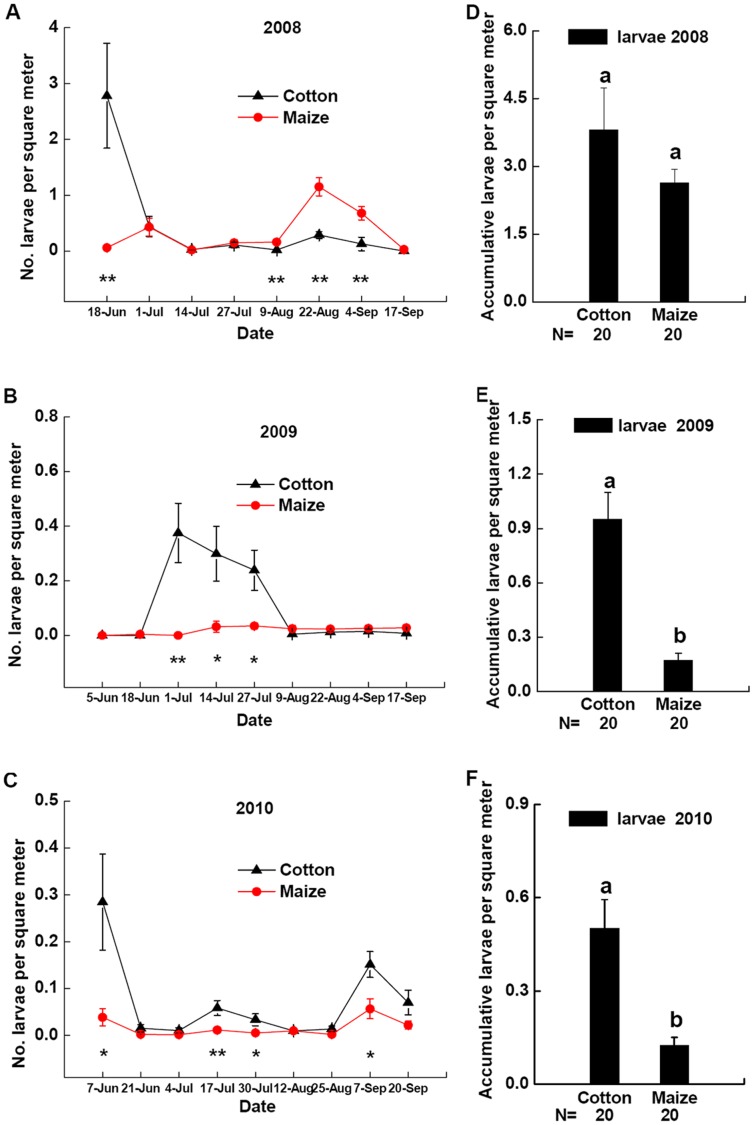
Dynamics of *P. japonica* larvae. Densities of *P. japonica* larvae in cotton patches (black triangle) and maize patches (red circle) in field landscape plots in 2008 (**A**), 2009 (**B**), and 2010 (**C**). *Significant differences between densities of *P. japonica* larvae in cotton patches and maize patches at p<0.05. **Significant differences between densities of *P. japonica* larvae in cotton patches and maize patches at p<0.01. Densities of accumulative *P. japonica* larvae in cotton patches and maize patches at all sample dates of field landscape plots in 2008 (**D**), 2009 (**E**) or 2010 (**F**). Different lowercases above the bars indicate significant differences in densities of accumulative *P. japonica* larvae in cotton patches and maize patches at p<0.05. Data are presented as adults per square meter of crop plants (mean±SE) with separate field landscape plots used as replicates. Sample size of cotton patch and maize patch are both 20. N indicates the size of samples tested.

### Laboratory Diet Experiments

Dietary shift experiment: Aphids from cotton and maize with distinct δ^13^C values were fed to larval and adult *P. japonica* to examine carbon isotope ratios of adult beetles and detect rates of change, if any, after a shift in the isotopic composition of the diet. Mature beetles were obtained from maize plants in the field at the Langfang Experiment Station. Eggs laid by mature beetles were placed into Petri dishes inside an environmental chamber, and 80 1^st^-instar larvae were raised on cotton aphids fed on cotton leaves until pupation occurred under the following conditions: 25°C with a photoperiod of L:D = 14∶10 and relative humidity of ∼80%. Upon emergence, a subsample of adult beetles (n = 6) was removed, labeled, placed in plastic vials containing 95% ethanol and stored in a freezer for preservation to serve as control samples. The remaining adults were fed on a diet of maize aphids that were fed on maize leaves in Petri dishes for 21 days. Subsamples of the remaining beetles (n = 6) were removed 1, 3, 5, 7, 14 and 21 days after the diet was changed to maize fed aphids. To establish the carbon isotope ratios of plants and aphids in the field, aphid and plant samples were also collected at the Langfang Experiment Station. Plant samples were cut from the upper leaves of cotton and maize plants, taking care to avoid the main leaf veins. Aphids were collected in groups of 20 or more by disturbing aphid colonies and collecting fallen or walking aphids with fine point forceps. As with adult *P. japonica* samples, aphid and plant material was preserved by freezing before sample preparation and analysis [5].

**Table 1:** Relationship between *P. japonica* eggs, larva and adults and and aphids in landscape plots during 2008, 2009 and 2010.^a^

No.	*P. japonica*	Year	Linear model^a^	R^2^	F	DF	P
**A**	Egg	2008	y = 0.8770×−2.5687	0.7028	14.1871	1,7	0.0093
		2009	y = 0.0504×−0.0769	0.8845	53.6286	1,7	0.0002
		2010	y = 0.0270×−0.0042	0.7212	18.1032	1,7	0.0038
**B**	Larva	2008	y = 0.3758×−1.1201	0.7320	16.3876	1,8	0.0067
		2009	y = 0.0322×−0.0287	0.8305	34.3064	1,8	0.0006
		2010	y = 0.0231×−0.0161	0.6335	12.0990	1,8	0.0103
**C**	Adult	2008	y = 0.2856×−0.7356	0.9743	227.2308	1,8	<0.0001
		2009	y = 0.0185×+0.0027	0.7367	19.5874	1,8	0.0031
		2010	y = 0.0519×+0.0040	0.5388	8.1776	1,8	0.0243

a x is the aphid density data, which was log-transformed (ln(n+1)) for analysis. y is density of *P. japonica* eggs, larva and adults.

Dietary proportion experiment: Larval and adult *P. japonica* were raised on a mixed diet of cotton and maize aphids at varying proportions to determine the effect on δ^13^C values in beetles feeding on a mixed diet in the field [29]. Five groups of *P. japonica* were grown from eggs to mature adults on diets composed of five different proportions of cotton and maize aphids. The proportions were based on the weight ratio of cotton to maize aphids and resulted in the following five diets based on the weight of the aphids: 100∶0, 75∶25, 50∶50, 25∶75 and 0∶100. Each test group consisted of 20 1^st^-instar beetle larvae, each placed in a Petri dish inside an environmental chamber. The mixed diet for each *P. japonica* was checked each day, and additional aphids were not added until the old diet had been entirely consumed. The beetles were reared in their respective treatments for 20 days until reaching maturity. Samples of mature adults from the five test groups were collected, labeled and placed into a freezer for preservation and analysis.

**Figure 5:**
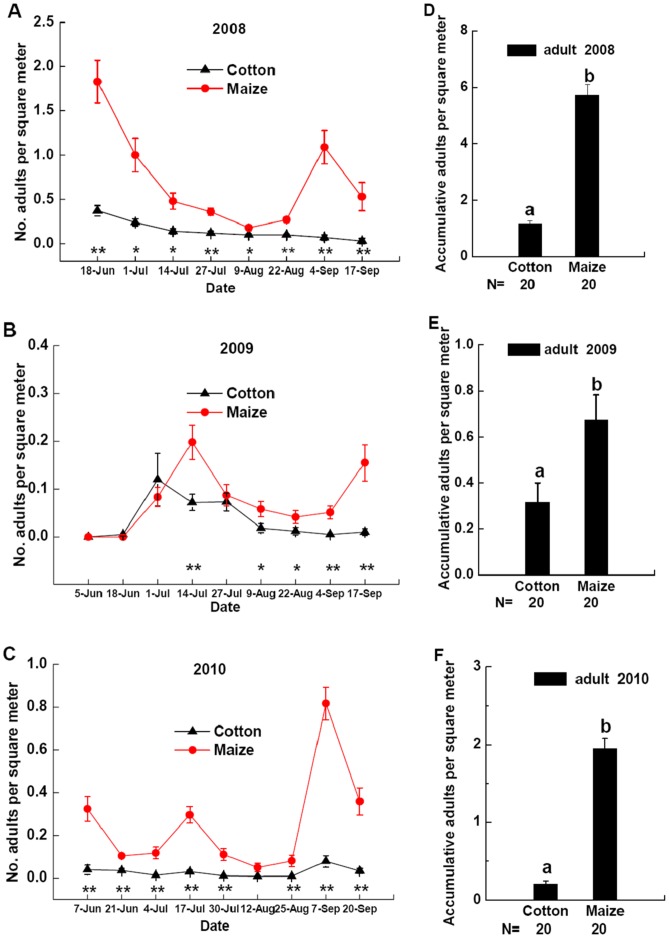
Dynamics of *P. japonica* adults. Densities of *P. japonica* adults in cotton patches (black triangle) and maize patches (red circle) in field landscape plots in 2008 (**A**), 2009 (**B**), and 2010 (**C**). *Significant differences between densities of *P. japonica* adults in cotton patches and maize patches at p<0.05. **Significant differences between densities of *P. japonica* adults in cotton patches and maize patches at p<0.01. Densities of accumulative *P. japonica* adults in cotton patches and maize patches at all sample dates of field landscape plots in 2008 (**D**), 2009 (**E**) or 2010 (**F**). Different lowercases above the bars indicate significant differences in densities of accumulative *P. japonica* adults in cotton patches and maize patches at p<0.05. Data are presented as adults per square meter of crop plants (mean±SE) with separate field landscape plots used as replicates. Sample size of cotton patch and maize patch are both 20. N indicates the size of samples tested.

**Table 2:** Repeated-measures analysis to population density of adult *P. japonica* between on two crops and among maize patches of various area in 2008, 2009 and 2010.^a^

Habitat	No.	Year	Source	DF	MS	F	Sig.
Two crops^b^	A	2008	Crop	1.0	25.9736	133.0928	<0.0001
			Time	2.5	11.3389	19.9779	<0.0001
			Crop×Time	2.5	6.2315	10.9791	<0.0001
	B	2009	Crop	1.0	0.1436	6.7609	0.0137
			Time	3.0	0.1695	9.9097	<0.0001
			Crop×Time	3.0	0.0808	4.7259	0.0039
	C	2010	Crop	1.0	3.7722	161.9092	<0.0001
			Time	2.8	1.9077	45.1971	<0.0001
			Crop×Time	2.8	1.3828	32.7600	<0.0001
Maize patches^c^	D	2008	Area	3.0	0.8689	2.7631	0.0760
			Time	3.2	14.4587	36.6108	<0.0001
			Area×Time	9.6	2.2248	5.6333	<0.0001
	E	2009	Area	3.0	0.0027	0.0866	0.9662
			Time	3.1	0.1464	8.9471	0.0001
			Area×Time	9.2	0.0145	0.8861	0.5467
	F	2010	Area	3.0	0.1945	13.0233	0.0001
			Time	2.9	3.1498	50.8075	<0.0001
			Area×Time	8.7	0.1525	2.4596	0.0230

a Statistic results corrected by Greenhouse–Geisser, as P value<0.05 (Mauehly’s Test of Sphericity).

b Two crops: cotton and maize.

c Maize patches of four area proportions: 25%, 50%, 75% and 100% of maize in plots.

### Sample Preparation and Stable Isotope Analysis

All samples collected and stored in a freezer for laboratory and field experiments were washed twice in reverse-osmosis filtered water. Accurate isotope analysis usually requires homogenization by grinding larger samples of solids into a fine powder and subsampling [5]. The forewings of each *P. japonica* adult were clipped and placed in an open 2.5-ml plastic vial under a stream of air to evaporate the residual alcohol and filtered water. The vial was then dried, capped and stored. Aphids were separated from the plant material with a scalpel and used in groups of approximately 50 individuals from ∼20 colonies. All of the samples were dried for 72 h at 65°C before being weighed to an accuracy of ±1 µg and packaged in tin sample capsules. Plant samples were large enough to require homogenization. After drying, leaf tissue was pulverized to a powder before enclosing a subsample of desired mass (2–3 mg) into a sample capsule.

**Figure 6:**
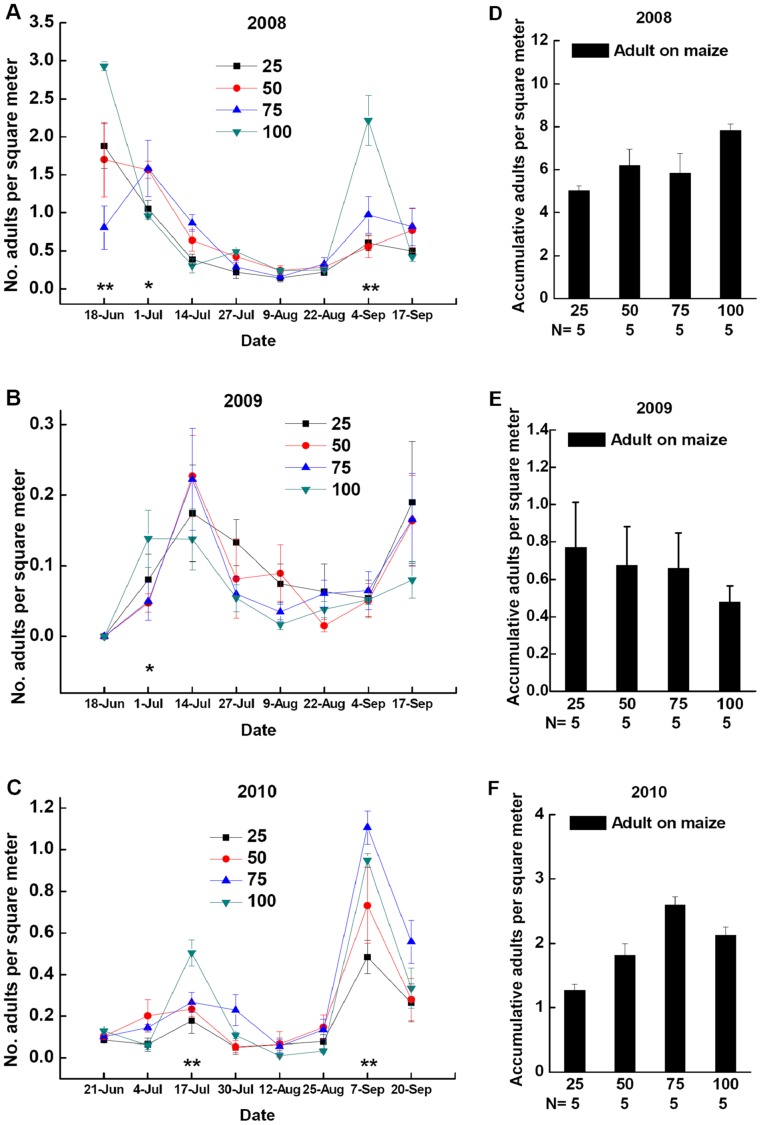
Dynamics of *P. japonica* adults on maize patches of four area proportions. Densities of *P. japonica* adults in maize patches of four area proportions: 25%, 50%, 75% and 100% of maize in field landscape plots in 2008 (**A**), 2009 (**B**), and 2010 (**C**). *Significant differences between densities of *P. japonica* adults in maize patches of four area proportions at p<0.05. **Significant differences between densities of *P. japonica* adults in maize patches of four area proportions at p<0.01. Densities of accumulative *P. japonica* adults in maize patches of four area proportions at all sample dates of field landscape plots in 2008 (**D**), 2009 (**E**) or 2010 (**F**). Different lowercases above the bars indicate significant differences in densities of accumulative *P. japonica* adults in maize patches of four area proportions at p<0.05. Data are presented as adults per square meter of crop plants (mean±SE) with separate field landscape plots used as replicates. Sample size of cotton patch and maize patch are both 5. N indicates the size of samples tested.

**Table 3:** Linear regression between densities of *P. japonica* adults on maize and landscape shape index of plot.^a^

Year	Date	Linear model	R^2^	F	DF	P
2008	18-Jun	y = −0.7802×+2.9926	0.1454	3.0620	1,18	0.0972
	1-Jul	y = −0.0733×+1.4002	0.0049	0.0882	1,18	0.7698
	14-Jul	y = −0.0830×+0.6729	0.0167	0.3061	1,18	0.5869
	27-Jul	y = −0.1843×+0.6320	0.2455	5.8561	1,18	0.0263
	9-Aug	y = −0.0344×+0.2496	0.0264	0.4887	1,18	0.4935
	22-Aug	y = 0.0743×+0.1620	0.0887	1.7527	1,18	0.2021
	4-Sep	y = −0.6581×+2.0681	0.1613	3.4624	1,18	0.0792
	17-Sep	y = 0.2969×+0.1853	0.1075	2.1672	1,18	0.1583
	Total b	y = −1.4420×+8.3627	0.1767	3.8629	1,18	0.0650
2009	18-Jun					
	1-Jul	y = −0.0294×+0.1286	0.0341	0.5640	1,16	0.4636
	14-Jul	y = 0.2131×−0.1312	0.5459	19.2371	1,16	0.0005
	27-Jul	y = 0.1086×−0.0811	0.3805	9.8267	1,16	0.0064
	9-Aug	y = 0.0983×+-0.0944	0.5562	20.0542	1,16	0.0004
	22-Aug	y = 0.0419×−0.0188	0.1370	2.5400	1,16	0.1306
	4-Sep	y = 0.0702×−0.0517	0.4584	13.5445	1,16	0.0020
	17-Sep	y = 0.1689×−0.1066	0.3039	6.9866	1,16	0.0177
	Total b	y = 0.6716×−0.3552	0.5915	23.1674	1,16	0.0002
2010	21-Jun	y = −0.0283×+0.1459	0.2189	5.0439	1,18	0.0375
	4-Jul	y = 0.0704×+0.0123	0.0972	1.9389	1,18	0.1808
	17-Jul	y = −0.0967×+0.4397	0.0827	1.6218	1,18	0.2190
	30-Jul	y = −0.0195×+0.1390	0.0067	0.1215	1,18	0.7314
	12-Aug	y = −0.0200×+0.0789	0.0145	0.2654	1,18	0.6127
	25-Aug	y = 0.0221×+0.0649	0.0115	0.2097	1,18	0.6525
	7-Sep	y = −0.2284×+1.1583	0.1192	2.4371	1,18	0.1359
	20-Sep	y = −0.0905×+0.4940	0.0343	0.6386	1,18	0.4346
	Total b	y = −0.3908×+2.5329	0.1205	2.4657	1,18	0.1338

a x is the landscape shape index of plot, y is the densities of *P. japonica* adults on maize patches of plot.

b the densities of *P. japonica* adults on maize patches in each plot were accumulated in all sample dates in 2008, 2009 or 2010.

Sample carbon isotope ratios were determined at the Chinese Academy of Forestry’s Stable Isotope Laboratory via a combustion-gas chromatography-mass spectrometry process. In this process the gases produced by flash combustion of samples are sent to a gas chromatograph where carbon dioxide (CO_2_) is separated out and sent to a mass spectrometer. The dried samples were placed in tin capsules and combusted to CO_2_ in a Flash EA1112 HT elemental analyzer. The δ^13^C measurements were made on a modified Finnigan MAT (Thermo Fisher Scientific, Inc., USA) Delta V advantage isotope ratio mass spectrometer. These isotope ratios are expressed as δ^13^C, where:
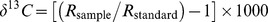
and the *R*_sample_ and *R*_standard_ are the ratios of ^13^C:^12^C for an individual sample and the analytical standard (Pee Dee Belemnite). This expresses isotope composition on a relative scale, and the use of an analytical standard allows for a conversion to absolute values.

### Statistical Analysis

This study was achieved with field and laboratory experiments. Results from field experiments show the densities of aphids and predatory beetle among cotton and maize patches. And data from laboratory experiments provided a method of stable carbon isotope analysis for field samples. All statistical analyses were conducted using SPSS software (SPSS.17, 2008). In the field experiments, treatments of spatial variation were set up by patch area and spatial arrangements of cotton and maize. Patch area was performed with one way ANOVA and with repeated measures of general lineal model. Spatial arrangements were analyzed with linear regression.

**Figure 7:**
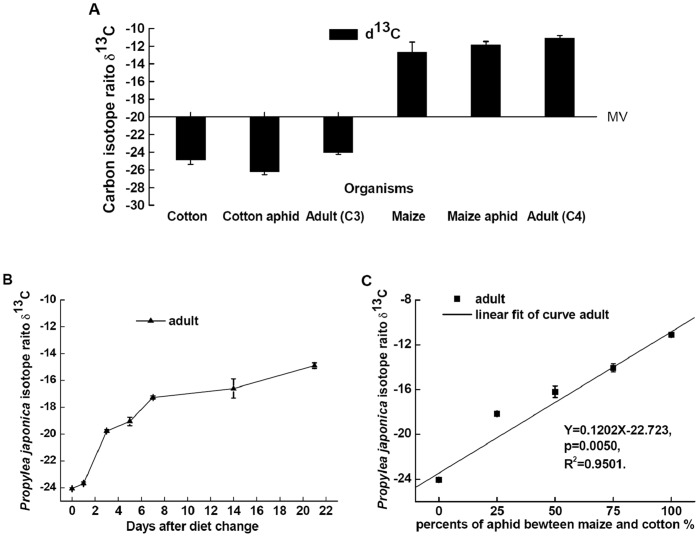
Carbon isotope ratios of *P. japonica* adults in laboratory. (**A**) Carbon isotope ratios of plant and insect species used in dietary switching experiment. Carbon isotope ratios (mean δ^13^±SD) of plants (cotton and maize), aphids from cotton and maize, and *P. japonica* adults. Adult (C3) was raised on cotton aphids. Adult (C4) was raised on maize aphids. MV, middle δ^13^C values of *P. japonica* adults was −16.71‰ as the proportion of aphids from a C_3_-based resource and a C_4_ -based resource was 50%. Error bars indicate the SD. (**B**) Carbon isotope ratios of *P. japonica* adults in dietary switching experiment. Carbon isotope ratios (mean δ^13^±SD) of laboratory-reared *P. japonica* adults (▴) before and after a shift in diet from a C_3_-based resource (cotton aphids reared on cotton) to one based on C_4_ plants (maize aphids reared on maize). (**C**) Relationship of δ^13^C values of *P. japonica* and proportions of aphids form C_3_ and C_4_-based resource. Aphids were from a C_3_-based resource and a C_4_ -based resource on which they were grown in the laboratory. The ladybird beetles were grown from eggs to adults on five food mixtures consisting of, respectively, 100% cotton aphids/0% maize aphids, 75% cotton aphids/25% maize aphids, 50% cotton aphids/50% maize aphids, 25% cotton aphids/75% maize aphids and 0% cotton aphids/100% maize aphids. Linear equation Y = 0.120X-22.723 (*F* = 57.08, *P* = 0.005, *R* ^2^ = 0.95): where Y is δ^13^ values of *P. japonica*, X is proportion of aphids from a C_3_-based resource and a C_4_-based resource.

To know oviposition preference of *P. japonica* within agricultural landscapes composed of cotton and maize, three ways were utilized. First, the densities of aphids, *P. japonica* eggs and larvae between on cotton and maize patches at each sample date were analyzed with one way ANOVA. Second, the densities of aphids, *P. japonica* eggs and larvae between on cotton and maize patches in each plot were accumulated in all sample dates in 2008, 2009 and 2010 respectively, and the densities of accumulative aphids, *P. japonica* eggs and larvae were also analyzed with one way ANOVA. Third, linear regression analysis was used to determine the relationship between *P. japonica* eggs, larvae and adults and aphids in landscape plots during 2008, 2009 and 2010.

**Figure 8:**
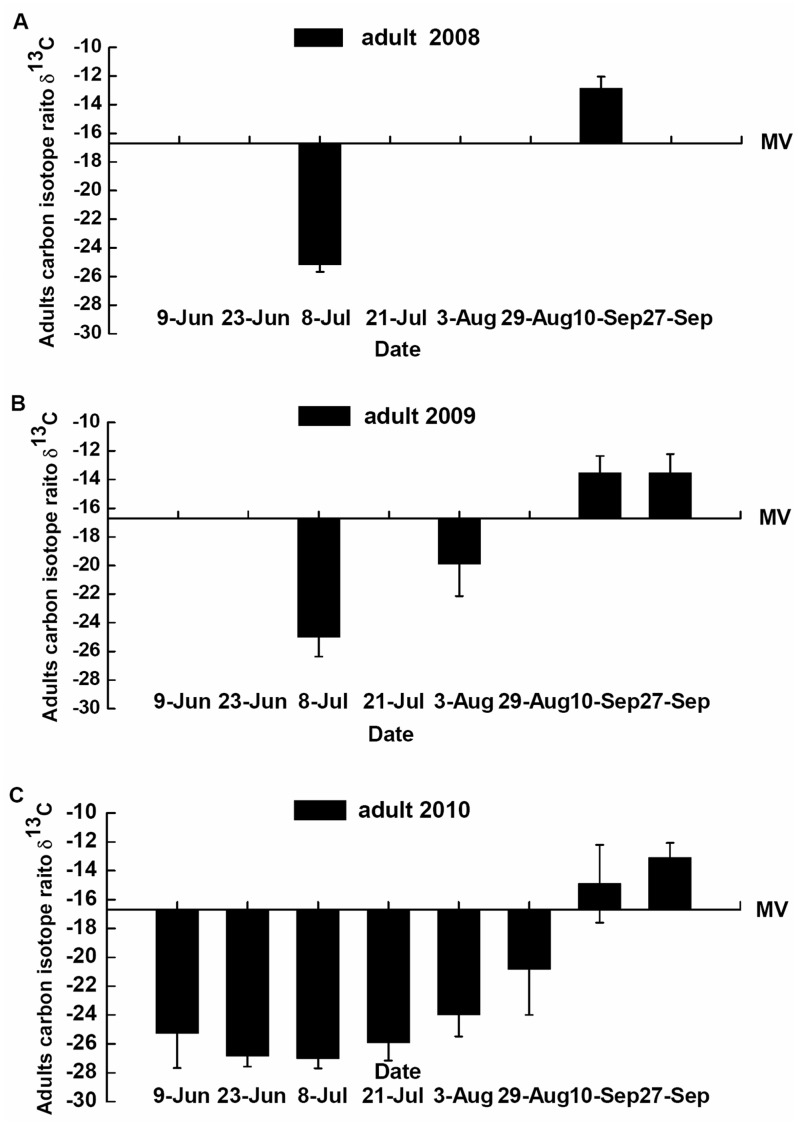
Carbon isotope ratios (mean δ^13^±SD) of *P. japonica* adults collected from maize patches. In 2008 (**A**), 2009 (**B**), 2010 (**C**). MV, middle δ^13^C values of *P. japonica* adults was −16.71 as the proportion of aphids from a C_3_-based resource (cotton aphids reared on cotton) and a C_4_ -based resource (maize aphids reared on maize) was 50%.

To identify the preferred crop patches of *P. japonica* adult, firstly, the densities of *P. japonica* adults between on cotton and maize patches at each sample date were analyzed with one way ANOVA. Secondly, the densities of *P. japonica* adults between on cotton and maize patches in each plot were accumulated in all sample dates, and the densities of accumulative *P. japonica* adults in 2008, 2009 and 2010 were also analyzed with one way ANOVA. Thirdly, effects of sample date (Time), crop type (Crop: cotton and maize) and interaction of both (Time×Crop) on the densities of *P. japonica* adults were tested with repeated measures of general lineal model. The responses of *P. japonica* adults on maize patches to spatial variation (maize patch area and spatial arrangement) were analyzed. Effect of maize patch area on the densities of *P. japonica* adults among maize patches of four area proportions (25%, 50%, 75% and 100% of maize in plots) were analyzed for three steps. First, the densities of *P. japonica* adults among maize patches of four area proportions at each sample date were analyzed with one way ANOVA. Second, the densities of *P. japonica* adults among maize patches of four area proportions in each plot were accumulated in all sample dates, and the densities of accumulative *P. japonica* adults among maize patches of four area proportions in 2008, 2009 and 2010 were also analyzed with one way ANOVA, and then multiple comparisons to maize patches of four area proportions were used with the Student-Newman-Keuls (SNK) test at α = 0.05. Third, effects of sample date (Time), maize patch area (Area) and interaction of both (Time×Area) on the densities of *P. japonica* adults were tested with repeated measures of general lineal model. The relationship between densities of *P. japonica* adults on maize and landscape shape index of plot was modeled with linear regression.

**Table 4:** Estimated proportion of diet for *P. japonica* adults originating from C_3_-based resources and/or C_4_-based resources in the field from 2008–2010.^a^

	2008		2009		2010	
Date of sample	C_3_-based resource	C_4_-based resource	C_3_-based resource	C_4_-based resource	C_3_-based resource	C_4_-based resource
**9-Jun**					**∼ 100%**	**∼ 0%**
**23-Jun**					∼100%	∼0%
**8-Jul**	∼100%	∼0%	∼100%	∼0%	∼100%	∼0%
**21-Jul**					∼100%	∼0%
**3-Aug**			76.51%	23.49%	∼100%	∼0%
**29-Aug**					84.20%	15.80%
**10-Sep**	17.91%	82.09%	23.53%	76.47%	34.84%	65.16%
**27-Sep**			23.55%	76.45%	19.89%	80.11%

a Proportions of diet for *P. japonica* adults were estimated based on the carbon isotope ratio linear equation and δ^13^C value of *P. japonica* adults collected in field.

For determining the feeding behavior of *P. japonica* in multiple crop landscapes, in the laboratory diet experiment, mean carbon isotope ratios (δ^13^C±SD) and shifts between trophic levels (**Δ** ***δ*** ^13^ **C**) were calculated for each sample type. The changes in beetle isotope ratios over time were presented graphically but not analyzed statistically. Regression analysis was used to determine the relationship between δ^13^C values of *P. japonica* adults and proportions of aphids from a C_3_-based resource (cotton aphids reared on cotton) and a C_4_-based resource (maize aphids reared on maize).

## Results

### Oviposition Preference: Dynamics of Aphids and *P. japonica* Eggs and Larvae

Aphid abundance was significantly different between maize and cotton patches in field landscape plots in 2008–2010 (Fig. 2 A, B, C). Aphid densities in cotton patches were greater than those in maize patches during some sample dates, while densities of aphids in maize patches were higher than those in cotton patches during in the other sample dates in 2008–2010 (Fig. 2 A, B, C). Densities of accumulative aphid in cotton patches were greater than those in maize patches during all the sample dates in 2008 (Fig. 2 D, *F* = 94.4349, *P*<0.0001) and 2009 (Fig. 2 E, *F* = 52.2376, *P*<0.0001), while densities of accumulative aphid in maize patches were greater than those in cotton patches in 2010 (Fig. 2 F, *F* = 4.3301, *P* = 0.0444).

Similarly, egg abundance of *P. japonica* was different between maize and cotton patches in field landscape plots in 2008–2010 (Fig 3 A, B, C). Densities of accumulative *P. japonica* eggs in cotton patches were greater than those in maize patches during all the sample dates in 2008 (Fig. 3 D, *F* = 4.7949, *P* = 0.0348) and 2009 (Fig. 3 E, *F* = 18.7710, *P* = 0.0001), while there was no significant differences in densities of accumulative *P. japonica* eggs between maize and cotton patches in 2010 (Fig. 3 F, *F* = 0.9634, *P* = 0.3327).

Larval abundance of *P. japonica* was also different between maize and cotton patches in field landscape plots in 2008–2010 (Fig 4 A, B, C). Densities of accumulative *P. japonica* larva in cotton patches were higher than those in maize patches during all the sample dates in 2009 (Fig. 4 E, *F* = 25.7596, *P*<0.0001) and 2010 (Fig. 4 F, *F* = 16.0858, *P* = 0.0003). Whereas densities of accumulative *P. japonica* larva in cotton patches was not significantly higher than those in maize patches during all the sample dates in 2008 (Fig. 4 D, *F* = 1.4271, *P* = 0.2396).

The relationships between densities of *P. japonica* eggs and larva and densities of aphids in agricultural landscapes can be described by a linear regression model (Table 1). Linear regression analysis revealed that egg densities of *P. japonica* were positively correlated with aphid densities in agricultural landscapes in 2008–2010 (Table 1 A). Similar results were found for regression analysis that larval densities of *P. japonica* were also positively correlated with aphid densities in agricultural landscapes in 2008–2010 (Table 1 B).

### Habitat Selection: Predatory Beetle Densities and the Relationship between *P. japonica* Adult and Aphid Densities

Repeated measures analysis indicated that *P. japonica* adult densities were significantly affected by crop type in field landscape plots composed of cotton and maize in 2008 (Table 2 A), 2009 (Table 2 B) and 2010 (Table 2 C). *P. japonica* adult densities established in maize patches were significantly greater than those in cotton patches during most of the sampling dates from 2008–2010 (Fig. 5 A, B, C). And densities of accumulative *P. japonica* adult in maize patches were significantly greater than those in cotton patches during all the sample dates in 2008 (Fig. 5 D, *F* = 133.8022, *P*<0.0001), 2009 (Fig. 5 E, *F* = 6.7601, *P* = 0.0137) and 2010 (Fig. 5 F, *F* = 161.9278, *P*<0.0001). *P. japonica* adult densities were significantly varied through time in 2008 (Table 2 A), 2009 (Table 2 B) and 2010 (Table 2 C). Moreover, the interaction of crop type and time were significant in 2008 (Table 2 A), 2009 (Table 2 B) and 2010 (Table 2 A). Linear regression analysis revealed that adult densities of *P. japonica* were positively correlated with aphid densities in agricultural landscapes in 2008–2010 (Table 1 C).

Based on the above results, the analysis focused on responses of *P. japonica* adults habitat in maize patches to spatial variation (maize patch area and spatial arrangement). Linear regression analysis revealed that densities of *P. japonica* adults on maize were not significantly correlated with landscape shape index of plot in 2008 and 2010 (Table 3). Repeated measures analysis indicated that *P. japonica* adult densities among maize patches of four area proportions were not significantly affected by maize patch area in field landscape plots in 2008 (Table 2 D) and 2009 (Table 2 E), however maize patch area significantly affected the densities of *P. japonica* adult in 2010 (Table 2 F). And densities of accumulative *P. japonica* adult among maize patches of four area proportions were not significantly affected by maize patch area in 2008 (Fig. 6 D, *F* = 2.7629, *P* = 0.0760) and 2009 (Fig. 6 E, *F* = 0.0866, *P* = 0.9662), but significantly affected in 2010 (Fig. 6 F, *F* = 13.0220, *P* = 0.0001). *P. japonica* adult densities among maize patches of four area proportions were significantly varied through time in 2008 (Table 2 D), 2009 (Table 2 E) and 2010 (Table 2 F). Moreover, the interaction of maize patch area and time were significant in 2008 (Table 2 D) and 2010 (Table 2 F), but not significant in 2009 (Table 2 E).

### Feeding Behavior: Changes in Feeding Behavior of *P. japonica* Adults as Indicated by Stable Carbon Isotope Analysis

#### 1. Changes in carbon isotope ratios following a dietary shift in the laboratory

Both groups of *P. japonica* control samples reflected the δ^13^ values of the diets of their aphid prey on which they were reared (Fig. 7 A). The δ^13^C values of cotton, cotton aphids and *P. japonica* adults from C_3_ resources were −25.6±0.5 (*n* = 15), −26.2±0.3 (*n* = 4) and −24.1±0.2 (*n* = 8), respectively. While, the δ^13^C values of maize, maize aphids and *P. japonica* adults from C_4_ resources were −12.7±1.1 (*n* = 9), −11.8±0.4 (*n* = 4) and −11.1±0.3 (*n* = 14), respectively (Fig. 7 A). Mean differences, or isotopic shifts (Δδ^13^C) based on C_3_ resources between trophic levels were −0.6‰ (cotton aphids to cotton), 2.1‰ (*P. japonica* to cotton aphids) and 1.5‰ (*P. japonica* to cotton), while mean differences, or isotopic shifts (Δδ^13^C) based on C_4_ resources between trophic levels were 0.9‰ (maize aphids to maize), 0.7‰ (*P. japonica* to maize aphids) and 1.6‰ (*P. japonica* to maize) (Fig. 7 A).

*P. japonica* δ^13^ values changed greatly after the original C_3_-based diet (cotton aphids) was changed to a C_4_-based resource (maize aphids), moving from −24.1±0.2‰ to −17.3±0.3‰ (mean±SD) in 7 days (Fig. 7 B). After feeding on cotton aphids exclusively for 21 days, the δ^13^ values of *P. japonica* reached −14.9±0.6‰, but were still diluted in ^13^C relative to their maize feeding aphid prey (−11.8±0.4‰) (Fig. 7 B).

The δ^13^C values for *P. japonica* adults in the dietary proportion experiment were −24.1±0.2‰ (n = 8; raised on a 100∶0 ratio of cotton aphids to maize aphids), −18.2±0.3‰ (*n* = 6; raised on a 75∶25 ratio of cotton aphids to maize aphids), −16.2±1.6‰ (*n* = 10; raised on a 50∶50 ratio of cotton aphids to maize aphids), −14.1±1.1‰ (*n* = 9; raised on a 25∶75 ratio of cotton aphids to maize aphids) and −11.1±0.3‰ (*n* = 14; raised on a 0∶100 ratio of cotton aphids to maize aphids) (Fig. 7 C). The regression of δ^13^C values (Y) between *P. japonica* adults and the proportion (X) of aphids from a C_3_-based resource (cotton aphids reared on cotton) and a C_4_ -based resource (maize aphids reared on maize) on which they were reared in the laboratory was expressed with a linear equation (Fig. 7 C), Y = 0.120X −22.723 (*F* = 57.08, *P* = 0.005, *R* ^2^ = 0.95 ).

Based on a carbon isotope ratio linear equation, the middle δ^13^C value of *P. japonica* adults was −16.71‰ when the proportion of aphids from a C_3_-based resource and a C_4_-based resource was 50%, which is in accordance with the −16.2±1.6‰ value observed for beetles raised from eggs to adults on the 50∶50 diet in the laboratory. In the experiment representing an agricultural landscape composed of C_3_ and C_4_ plants, if the δ^13^C value of adult beetles was below −16.71‰ the diet was assumed to consist of greater than 50% cotton aphids, whereas a value above −16.71‰ indicated the diet included more than 50% maize aphids.

#### 2. Feeding changes of adults in carbon isotope ratios collected in the field

Three hundred and four samples of *P. japonica* adults collected from maize patches in the field were analyzed from 2008−2010. The δ^13^C values (mean±SD) of *P. japonica* adults collected on 8-Jul and 10-Sep-2008 (Fig. 8 A) were −25.2±0.5 (*n* = 20) and −12.9±0.8 (*n* = 20), respectively. The δ^13^C values (mean ± SD) of *P. japonica* adults collected on 8-Jul, 3-Aug, 10-Sep and 27-Sep-2009 (Fig. 8 B) were −25.0±1.4 (*n* = 42), −19.9±2.2 (*n* = 20), −13.5±1.2 (*n* = 20) and −13.5±1.3 (*n* = 19), respectively. The δ^13^C values (mean ± SD) of *P. japonica* adults collected on 9-Jun, 23-Jun, 8-Jul, 21-Jul, 3-Aug, 29-Aug, 10-Sep and 27-Sep-2010 (Fig. 8 C) were −25.3±2.4 (*n* = 24), −26.8±0.7 (*n* = 20), −27.0±0.7 (n = 20), −25.9±1.2 (*n* = 20), −24.0±1.5 (*n* = 20), −20.8±3.2 (*n* = 19), −14.9±2.7 (*n* = 20) and −13.1±1.3 (*n* = 20), respectively.

Based on the carbon isotope ratio linear equation (Fig. 7 C) and the middle δ^13^C values of *P. japonica* adults (MV = 16.71‰), the mean δ^13^C value of *P. japonica* adults collected on 8-Jul was lower than the MV, but the mean value for beetles collected on 10-Sep-2008 was higher than the MV (Fig. 8 A). Mean δ^13^C values of *P. japonica* adults collected on 8-Jul and 3-Aug were lower than the MV, while those collected on 10-Sep and 27-Sep-2009 were higher than the MV (Fig. 8 B). The mean δ^13^C values of *P. japonica* adults collected from 9-Jun to 29-Aug were lower than the MV, whereas those collected on 10-Sep and 27-Sep-2010 were higher than the MV (Fig. 8 C).

According to the δ^13^C values (Fig. 8) and the linear equation (Fig. 7 C), the estimated diet proportions for *P. japonica* adults in the field from 2008–2010 were given (Table 4). In 2008, the diet of *P. japonica* adults collected on 8-Jul was estimated at ∼100% from C_3_-based resources, whereas 82.09% of the diet came from C_4_-based resources for beetle adults collected on 10-Sep. In 2009, the diets for *P. japonica* adults collected on 8-Jul and 3-Aug were estimated to consist of ∼100% and 76.51% C_3_-based resources, respectively, while 76.47% and 76.45% of the diet originated from C_4_-based resources for adults collected on 10-Sep and 27-Sep. In 2010, ∼100% of the diet for *P. japonica* adults collected from 8-Jul to 3-Aug came from C_3_-based resources and 84.20% of the diet for adults collected on 29-Aug came from C_3_-based resources. Nevertheless, 65.16% and 80.11% of the diet for adults collected on 10-Sep and 27-Sep came from C_4_-based resources.

## Discussion

Habitat management, a form of conservation biological control, is an ecologically based approach aimed at creating a suitable ecological infrastructure within the agricultural landscape to favor natural enemies by providing resources such as food for larval or adult natural enemies, alternative prey or hosts, and shelter from adverse conditions [4]. Our results from this three-year study found that *P. japonica* adults actively search host plants for aphids before ovipositing, regardless of the composition of the agricultural landscape as evidenced by *P. japonica* egg densities being positively correlated with aphid densities rather than host plant. The data indicate that the predatory beetle seeks out high prey densities before ovipositing; presumably this is to ensure there is enough food for their offspring. Densities of *P. japonica* adults in maize patches were significantly greater than those in cotton patches during most of sampling dates in the agricultural systems. Adults of *P. japonica* apparently prefered to inhabit maize patches, even when prey was scarce in maize and abundant in cotton. *P. japonica* adults in maize were not significantly positively correlated with aphids in maize, whereas they were significantly positively correlated with aphids in cotton and in landscape plots in agricultural systems. In the landscape plots, *P. japonica* adults were significantly positively correlated with aphids. The results imply that *P. japonica* adults preferentially inhabit maize patches but that they will transfer from cotton patches to forage on cotton aphids in agricultural systems consisting of both transgenic cotton and maize crops. Maize may serve as a better habitat or shelter for the predatory beetle from adverse conditions in agricultural landscapes composed of transgenic cotton and maize.

Before planting of cotton and maize, organic fertilizer was used in experimental field. One part of the organic fertilizer was composed of maize straw and leaf, and eggs of maize aphid may be laid on maize straw and leaf to overwinter. More organic fertilizer composed of maize straw and leaf was used in 2010 than in 2008 and 2009. Thus, more overwintering eggs of maize aphid were took into the field in 2010 than in 2008 and 2009 at early stage of plant. Aphid densities in maize patches were more than in cotton patches during 2010 on 7 Jun (Fig. 2 C), and then densities of accumulative aphid in maize patches were greater than those in cotton patches in 2010 (Fig. 2 F). The variance among years may be related to the dispersal abilities of adult beetle, spatial structure of adult beetles and also climate conditions. In fact, we have analyzed the effects of biological control of the beetles on aphids. However, the densities of aphids on cotton patches among these plots with different landscape design (ratio of cotton to maize) were not significantly affected by cotton patch area in field landscape plots during 2008–2010 at most instance. And the densities of aphids on maize patches were also not significantly affected by maize patch area in field landscape plots during 2008–2010 at most instance. The natural control effect of the beetles on aphids was no significant among these plots with different landscape design, and the reason may be that the beetles can disperse among these plots at large spatial scale.

Carbon isotope ratio analyses have been widely used for better assessment of alternate host use by *Helicoverpa zea* [30], crop colonization, feeding, and reproduction by the predatory beetle, *Hippodamia convergens* [5] and dispersal abilities of adult click beetles, *Agriotes obscurus*, in arable land [16]. The δ^13^C values of adult *P. japonica* in a dietary shift experiment documented that individual beetles shifting from a C_3_- to a C_4_-based diet of aphids reared on maize or cotton, respectively, would start to reflect the isotope ratio of their new C_4_ resources within one week if adequate food was available. Similar results were obtained in a dietary shift experiment on the related *Hippodamia convergens*, which showed a distinct transition towards the δ^13^C value of its new diet 1–14 days after the isotopic composition of its diet was changed, and reflected the isotope ratio of their new C_3_ resources within 2 weeks [5]. The time needed for the δ^13^C value of lady beetle carbon to change after a dietary shift from C_3_ to C_4_ resources, or vice versa, was different between these two species of predatory beetles, possibly due to different assimilation rates of ingested carbon.

The dietary proportion experiment used the carbon isotope ratio linear equation to quantify the relative contribution of C from different plants at the base of the food web. The diet of adult *P. japonica* in the field can be distinguished between C_3_ and C_4_ resources from the carbon isotope ratio linear equation, and the proportion of C_3_ and C_4_ resources ingested could be estimated when *P. japonica* preyed on both cotton and maize aphids with distinct δ^13^C values within approximately 2 weeks. Similar data showed that the δ^13^C values of whole body samples of the beetle *Tribolium castaneum* were closely correlated with the δ^13^C values of its diet consisting of various proportions of maize and wheat [29]. Likewise, a mass balance equation can also be used to confirm the relative contribution of C from different plants at the base of the food web, estimate the magnitude of the change in δ^13^C values between trophic levels and verify that isotope ratios of consumers are sensitive to changes in their food source [17]. In the present study, the carbon isotope ratio linear equation was acquired on the basis of five proportions of maize and cotton diets, while the mass balance equation has typically been obtained using either 100% maize or 100% cotton. Therefore, the carbon isotope ratio linear equation is preferred for quantifying the proportional contribution from C_3_ and C_4_ resources in this agricultural system.

The data calculated by the carbon isotope ratio linear equation exhibited that approximately 80–100% of the adult *P. japonica* diet in maize patches originated from C_3_-based resources in June, July and August, while approximately 80% of the diet originated from C_4_-based resources in September. This result directly reflects that *P. japonica* adults may seek refuge in maize patches that are absent of its prey but actively search and feed on cotton aphids in the cotton patches throughout June, July and August. Therefore, in agricultural systems consisting of cotton and maize, maize can serve as a favorable habitat of *P. japonica* adults, and transgenic cotton often heavily infested with aphids, can offer an abundant food source for the predatory beetle. According to our previous research, approximately 5,356, 3-day-old aphids were consumed by *P. japonica* during the adult stage [14], which indicates that this mobile predatory beetle inhabiting maize patches can enhance the greater capacity to naturally control cotton aphids in the agricultural system.

In conclusion, besides serving as a refuge from selection pressure for adaptation to transgenic cotton varieties that produce a toxin from the bacterium *Bacillus thuringiensis* for *Helicoverpa zea* [30], maize can provide a favorable habitat for natural enemies capable of controlling cotton aphids on transgenic Bt cotton in an agricultural system composed of cotton and maize. In many parts of the world, transgenic crops such as Bt crops have come to dominate agricultural landscapes, which has often led to non-target insect pests becoming the key pests within cotton and many other non-Bt host crops [10], [11]. Recent studies report that the decrease in use of insecticide sprays associated with Bt crops could enhance biocontrol services, and found evidence that the predators might provide additional biocontrol services spilling over from Bt cotton fields onto neighbouring crops (maize, peanut and soybean) [31]. However our results clearly indicate that *P. japonica* adults lay their eggs on aphid infested host plants in large agricultural fields, preferentially inhabit maize patches, but move to cotton patches to feed on aphids, which suggests that habitat management with a suitable proportion and spatial arrangement of cotton and maize may be an alternative planting pattern to enhance biological control in order to meet the challenge of managing non-target pest densities in Bt cotton. Furthermore, maize patch area and spatial arrangement did not significantly effected *P. japonica* adult densities among maize patches in most instances. This results indicated *P. japonica* adult widely inhabit in maize patches in landscape field of 90 m×90 m. Maize benefits predators to provide potential to enhance biological control for non-target pests in transgenic cotton in field, while further work is needed to determine how to maintain and enhance biological control for insect pests in larger region for long time in agricultural landscape systerm.
